# Use of microspheres in embolization for unruptured renal angiomyolipomas

**DOI:** 10.1515/med-2021-0280

**Published:** 2021-04-20

**Authors:** Masashi Shimohira, Keiichi Nagai, Kengo Ohta, Yusuke Sawada, Taku Naiki, Takashi Nagai, Takahiro Yasui, Yuta Shibamoto

**Affiliations:** Department of Radiology, Nagoya City University Graduate School of Medical Sciences, Nagoya 467-8601, Japan; Department of Nephro-urology, Nagoya City University Graduate School of Medical Sciences, Nagoya, Japan

**Keywords:** renal angiomyolipoma, microspheres, embolization

## Abstract

**Purpose:**

To describe our initial experience with use of microspheres in transcatheter arterial embolization (TAE) for unruptured sporadic renal angiomyolipomas (AMLs).

**Materials and methods:**

Seven consecutive patients with seven unruptured sporadic renal AMLs, 6 females and 1 male, with a median age of 45 years (range, 30–69 years), underwent TAE using microspheres between November 2016 and February 2020. We evaluated the technical success rate, complications related to the procedure, clinical success rate, and the shrinkage rate of renal AML. Technical success was defined as the completion of TAE. Clinical success was defined as presence of shrinkage of the renal AML after TAE.

**Results:**

In all patients, TAE using microspheres was accomplished and technical success rate was 100% (7/7). Three patients exhibited slight pain, but it improved with only observation, and the minor complication rate was 43% (3/7) and major complication rate was 0% (0/7). After the TAE, shrinkage of renal AML was confirmed in 6 of 7 patients, and clinical success rate was 86% (6/7). The median of shrinkage rate was 47% (range, 26–83%) with a median follow-up period of 19 months (range, 4–30 months).

**Conclusion:**

TAE using microspheres appears to be effective and safe for unruptured sporadic renal AMLs.

## 1 Introduction

Renal angiomyolipomas (AMLs) are benign tumors composed of differing degrees of fat, smooth muscle, and abnormal blood vessels and have a propensity to bleed [1,2]. Transcatheter arterial embolization (TAE) is an important treatment for renal AMLs [3,4]. Ethanol has been widely used as embolic material in TAE [5,6]. However, disadvantages of ethanol embolization include difficulty to control placement, rapid dilution by vascular inflow, and severe pain [7]. Polyvinyl alcohol (PVA) particles were also widely used to embolize renal AMLs [8,9,10], but they aggregate easily due to their irregular shape and size variability [11]. Vessels might be occluded more proximally than intended, and it can even cause a microcatheter obstruction. On the other hand, microspheres have recently become available in our country. They are precisely calibrated by size, and smoother and more spherical in shape, without fragmentation, than PVA particles [7]. This prevents particle aggregation, thereby allowing the microspheres to better penetrate into smaller vessels than PVA particles of the same size. In this report, we describe our initial experience with the use of microspheres in TAE for unruptured sporadic renal AML.

## 2 Materials and methods

This retrospective study was approved by the Institutional Review Board of Nagoya City University Graduate School of Medical Sciences (approval number 60-19-0205). Written informed consent for the procedure had been obtained from each patient. Seven consecutive patients with seven unruptured sporadic renal AMLs, 6 females and 1 male, with a median age of 45 years (range, 30–69 years), underwent TAE using microspheres between November 2016 and February 2020. The indicative criteria for TAE were 4 cm or larger, or had 5 mm or larger aneurysmal formation [3].

**Figure 1:**
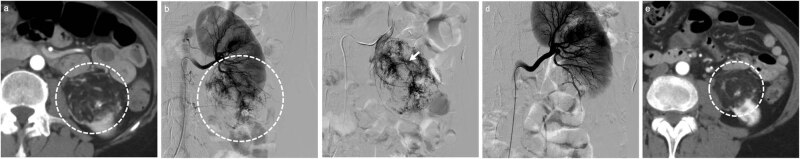
A 56-year-old woman with left unruptured sporadic renal AML. (a) Contrast-enhanced CT showed AML in the left kidney (circle). The size of the AML was 56 × 48 mm. (b) Enhancement of the AML (circle) was confirmed by angiography. (c) A microcatheter was advanced into the feeding artery of the AML (arrow). Thereafter, TAE was performed through the microcatheter using 100–300 μm microspheres. (d) Disappearance of enhancement of the tumor was confirmed. The renal infarction rate was <10%. Slight pain occurred, but it improved with observation. (e) Contrast-enhanced CT 30 months after TAE showed shrinkage of the AML and the size was 44 × 33 mm (circle). The shrinkage rate was 46%.

We reviewed medical records and images and evaluated the technical success rate, complications related to the procedure, clinical success, and the shrinkage rate of renal AMLs. Technical success was defined as the completion of TAE. Complications that required extended hospitalization, required an advanced level of care, or resulted in permanent adverse sequelae or death were classified as major complications, and the remaining complications were considered minor [12]. When focal renal infarction was found, the infarction rate was categorized into: <10%, 10–20%, and >30% using angiography immediately after TAE and follow-up computed tomography (CT) according to previously reported criteria [13]. Clinical success was defined as the presence of shrinkage of the renal AML after TAE. Areas of AML were calculated with the following formula [14] from axial CT images on slices showing the maximum AML diameter: (long-axis length × short-axis length) × (π/4). The shrinkage rate of AML was calculated with the following formula: {(initial area − follow-up area)/initial area} × 100 (Figure 1a and e). These images were interpreted by two radiologists with more than 13 years of experience in diagnostic and interventional radiology. Any discrepancies were resolved by consensus.

### 2.1 Technique of TAE using microspheres for renal AML

All procedures were approached via the common femoral artery. A 4-Fr sheath was introduced, followed by a 4-Fr catheter. The 4-Fr catheter was advanced to the renal artery, and angiography was performed to confirm the feeding artery and the stain of the renal AML (Figure 1b). A microcatheter was then advanced into the feeding artery of the renal AML as close as possible. Microspheres (Embosphere; Nippon Kayaku, Tokyo, Japan) were suspended in contrast media diluted with normal saline and then injected until the stain of renal AML disappeared (Figure 1c and d). When there were multiple feeding arteries, they were embolized in the same manner. Other embolic materials were not used. In all patients, an intravenous drip infusion of 15 mg pentazocine in 100 mL saline was administered for pain control during TAE, and the same regimen was added when pain occurred after TAE.

## 3 Results

Results of the TAE using microspheres for renal AMLs are summarized in Table 1. All patients did not have any symptom. The median size of the AML was 54 mm (range, 40–102 mm), and there was no aneurysmal formation. In all patients, TAE using microspheres was accomplished and thus technical success rate was 100% (7/7). Microspheres of 100–300 µm were used in all patients. Three patients exhibited slight pain, but it improved with only observation. In no patient, extended hospitalization or an advanced level of care was necessary, and no permanent adverse sequelae or death occurred. Thus, the minor complication rate was 43% (3/7) and major complication rate was 0% (0/7). There was focal renal infarction in 3 of 7 patients (43%), but infarction rate was less than 10% in all patients. It was confirmed that there was no renal dysfunction by blood examination. After the TAE, shrinkage of renal AML was confirmed in 6 of 7 patients, and thus clinical success rate was 86% (6/7). The median of shrinkage rate was 47% (range, 26–83%) with a median follow-up period of 19 months (range, 4–30 months). In one patient (14%), the shrinkage of renal AML was not obtained at the follow-up CT 7 months after the TAE, and repeat TAE was performed with ethanol and then the AML was shrunk.

**Table 1:** Results of the TAE using microspheres for renal AMLs

Case no.	Age	Sex	Location	Size (mm)	Technical success	Complication	Renal infarction	Follow-up period (Mo)	Shrinkage rate (%)	Clinical success	Re-TAE
1	56	F	L	56	Yes	Flank pain	<10%	30	46	Yes	No
2	32	F	L	40	Yes	No	No	26	72	Yes	No
3	45	F	L	86	Yes	No	No	24	26	Yes	No
4	45	M	R	51	Yes	Flank pain	<10%	14	48	Yes	No
5	39	F	L	40	Yes	Flank pain	<10%	12	83	Yes	No
6	69	F	R	102	Yes	No	No	4	36	Yes	No
7	30	F	L	54	Yes	No	No	7	0	No	Yes

TAE; transcatheter arterial embolization, AML; angiomyolipoma, L; left kidney, R; right kidney.

## 4 Discussion

In this study, we demonstrated a high technical success rate (100%) and a clinical success rate (86%) with low complication rates (minor 43%, major 0%). They were acceptable in comparison with those of literature [5,10,15,16].

Ethanol is common embolic material to embolize renal AMLs. It denatures blood proteins, clumps damaged erythrocytes, dehydrates vascular endothelial cells, and denudes the vascular wall of all endothelial cells [17]. The advantage of ethanol is that it causes permanent occlusion at a capillary level, thereby inducing tissue necrosis, making it a strong embolic agent. However, there is an associated risk of reflux and nontarget embolization inducing renal infarction. It was recently reported that use of micro-balloon catheter can prevent reflux of ethanol and strengthen the effects of ethanol by preventing dilution from the blood flow and contribute to decreasing the amount of ethanol [18]. Meanwhile, the limitation of the micro-balloon catheter is its high cost, at approximately US$1,000.

In this study, focal renal infarction was found in 43%, but they were less than 10%, and no patients had renal dysfunction. Microspheres are compressible and have little tendency to clump together and they can pass through a microcatheter easily and reach the distal vessels corresponding to the particle size. It can contribute to targeted embolization with minimum damage of the normal tissue [11]. Besides, for patients with alcohol hypersensitivity, it can be an important alternative option. In literature, size of used PVA particles for renal AMLs was variously reported to be 47–90 μm to 500–710 μm [8,10,15]. In the present study, we showed use of 100–300 μm size of microspheres with good results, and small size particle may contribute to deep penetration of microspheres into the tumor vasculatures.

One patient of this study had no shrinkage at the 7-month follow-up and ultimately required repeat TAE. We suspect that this was due to a technical fault, such as rapid injection of microspheres leading to particle aggregation and proximal embolization. Therefore, the renal AML might not be embolized sufficiently. Hence, microspheres should be injected very slowly to prevent particle aggregation. On the other hand, rupture of the AML during TAE using PVA particles was previously reported [9]. This could be attributed to an increase in intra-tumor pressure. This complication needs to be considered even in TAE using microspheres.

On the other hand, surgery can also be a treatment option for the renal AMLs. However, it has a high risk of bleeding, particularly when the AML is large [19]. Besides, antiplatelet drug therapy can increase a risk of intraoperative bleeding [20]. Thus, it is important to reduce the intraoperative blood loss. TAE has been recently reported useful for reduction of intraoperative blood loss for complex renal tumors [21]. It may also contribute to clear surgical margins [22]. Therefore, we think TAE can be an important procedure even in surgery for the renal AML.

The present study has several limitations. The retrospective design was key limitation. Sample size was small and the follow-up period was relatively short, and thus further investigation should be needed with large sample size and long follow-up period. More than one operator performed the TAEs, and the procedure was not standardized due to variability in the technique according to individual operator.

## 5 Conclusion

TAE using microspheres appears to be effective and safe for unruptured sporadic renal AMLs.

## Abbreviations

AML: angiomyolipoma
CT: computed tomography
PVA: polyvinyl alcohol
TAE: transcatheter arterial embolization
